# Nicotinamide Phosphoribosyltransferase May Be Involved in Age-Related Brain Diseases

**DOI:** 10.1371/journal.pone.0044933

**Published:** 2012-10-11

**Authors:** Li-Ying Liu, Feng Wang, Xia-Yan Zhang, Peng Huang, Yun-Bi Lu, Er-Qing Wei, Wei-Ping Zhang

**Affiliations:** 1 Department of Pharmacology, Zhejiang University School of Medicine, Hangzhou, Zhejiang Province, China; 2 Key Laboratory of Medical Neurobiology of Ministry of Health of China, Zhejiang University School of Medicine, Hangzhou, Zhejiang Province, China; 3 Zhejiang Province Key Laboratory of Neurobiology, Zhejiang University School of Medicine, Hangzhou, Zhejiang Province, China; University of New South Wales, Australia

## Abstract

Nicotinamide phosphoribosyltransferase (NAMPT) is a key enzyme for nicotinamide adenine dinucleotide (NAD) biosynthesis, and can be found either intracellularly (iNAMPT) or extracellularly (eNAMPT). Studies have shown that both iNAMPT and eNAMPT are implicated in aging and age-related diseases/disorders in the peripheral system. However, their functional roles in aged brain remain to be established. Here we showed that upon aging, NAMPT level increased in serum but decreased in brain, decreased in cortex and hippocampus but remained unchanged in cerebellum and striatum in brain, and increased in microglia but likely decreased in neuron. Accordingly, total NAD (tNAD) level significantly decreased in hippocampus, cerebellum and striatum in aged brain. Application of recombinant NAMPT, mimicking the elevated serum NAMPT level, enhanced the susceptibility of cerebral endothelial cells to ischemic injury, while inhibition of iNAMPT by FK866, a specific inhibitor, reduced intracellular NAD level and induced neuronal death. Taken together, we have revealed a region- and cell-specific change of NAMPT level in brain and serum upon aging, deduced its potential consequences, which suggests that NAMPT is a regulatory factor in aging and age-related brain diseases.

## Introduction

Nicotinamide phosphoribosyltransferase (NAMPT) is a key enzyme for synthesizing nicotinamide adenine dinucleotide (NAD) [1]. It is expressed in many different organs and tissues including brain [2], [3], liver [4], bone marrow [5], skeletal muscle [6] and adipose tissue [7]. NAMPT can be found either intracellularly (iNAMPT) or extracellularly (eNAMPT) [8]. iNAMPT participates in the salvage pathway of NAD synthesis — NAD plays a vital role in energy metabolism, serving as a cofactor of histone deacetylase sirtuins, regulates cell death through poly(ADP-ribose) polymerase 1 (PARP-1) [9], thus linking iNAMPT to these important cellular processes [10].

eNAMPT, mostly in the form of serum NAMPT, likely functions as a cytokine in circulation. Historically, it has been named pre-B cell enhancing factor (PBEF), as it was first cloned from human peripheral blood lymphocyte and could enhance B cell maturation [5]. eNAMPT has also been named visfatin, as it was found released from adipocytes and participated in energy homeostasis [7]. It has also been proposed that eNAMPT may function as an enzyme and synthesize NAD. However, due to the scarcity of adenosine-triphosphate (ATP) in the extracellular space, the enzymatic activity of eNAMPT was poor under normal circumstances [11].

NAMPT is closely related to the aging process. It can be beneficial for anti-aging and was found to slow down aging processes in several eukaryotic organisms, through synthesizing NAD and increasing the activity of sirtuins [12]–[15]. It has also been reported that the cellular lifespan could be extended by increasing the expression of NAMPT [16]. On the other hand, NAMPT can also assist the progress of aging process, and has been implicated in many age-related disorders and diseases including obesity [17], diabetes [18], cancer [19], inflammatory [20] and cardio-cerebra-vascular diseases [21]. In particular, eNAMPT has been found mediating immune responses, inflammation and oxidative responses [20], [22], [23], and participating in many age-related disorders [21], [24]. As such, the effect of NAMPT on aging and cellular processes is double-edged, which depends on its expression level and distribution.

In brain, iNAMPT is mainly expressed in neurons [2], [3], thus to meet the large energy demand, as neuron accounts for ∼70% of oxidative metabolism in cortical gray matter while brain accounts for ∼20% of total body oxygen consumption [25]. Upon aging, energy metabolism in neuron declines while the activity of microglia increases [26], [27].On the other hand, serum NAMPT has been implicated in many age-related peripheral disorders and diseases [17], [21], [28] and may cross the brain-blood barrier under certain conditions. Yet, during normal aging process (a non-diseased state), it remains to be characterized how brain iNAMPT and serum NAMPT level changes and whether such change is involved in brain aging. To this end, here we characterized NAMPT expression and distribution in serum and in brain, measured the relative NAD production in brain regions, and evaluated the effect of NAMPT alteration on the viability of cerebral vascular endothelial cells and neurons.

## Results

### Quantification of NAMPT protein level

**Figure 1:**
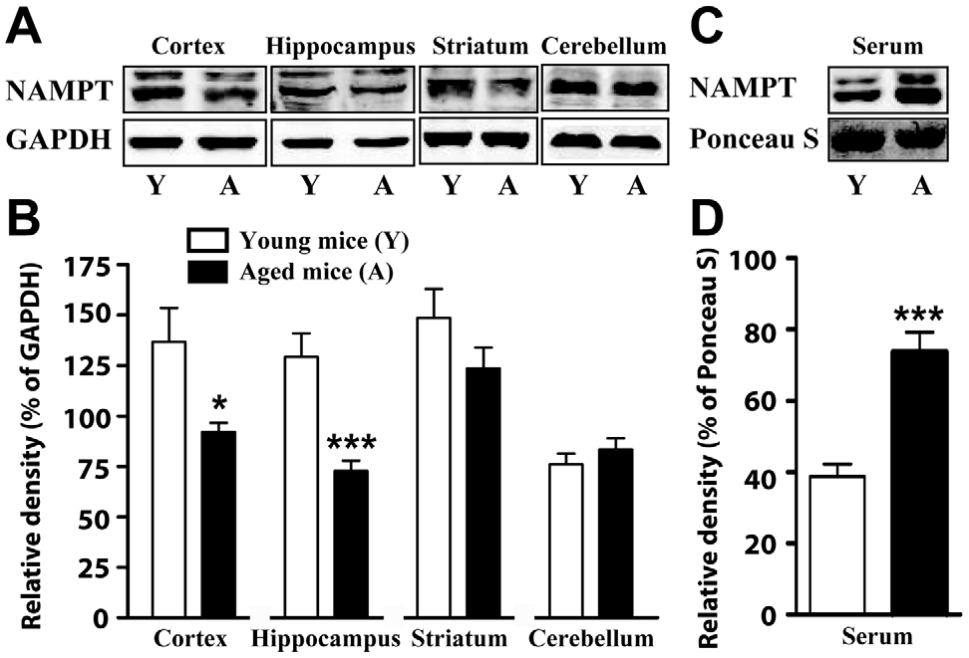
NAMPT expression in blood serum and brain regions for young and aged mice. A) Representative immunoblots of NAMPT and GAPDH expression in cortex, hippocampus, striatum and cerebellum. B) Statistical analyses of NAMPT expression in A. C) Representative immunoblots of NAMPT expression and ponceau staining in blood serum. D) statistical analyses of NAMPT expression in C. N = 10 for young mice and 11 for aged mice. Mean ± SEM. *P<0.05, ***P<0.001, compared with young mice, unpaired *t* test.

Using Western blot, we determined NAMPT protein level in serum and four brain regions including cortex, hippocampus, striatum and cerebellum. Total NAMPT level was found to be significantly lower in cortex and hippocampus regions of aged mice than that of young mice, but showed no significant differences in striatum and cerebellum (Fig. 1A and 1B). Whereas, there was no significant difference between NAMPT expression in middle-aged and young mice brain (Figure S1). On the other hand, eNAMPT level was significantly higher in the serum of aged mice and middle-aged mice than that of young mice (Fig. 1C and 1D, Figure S1).

### Tissue tNAD and NADH level

**Figure 2:**
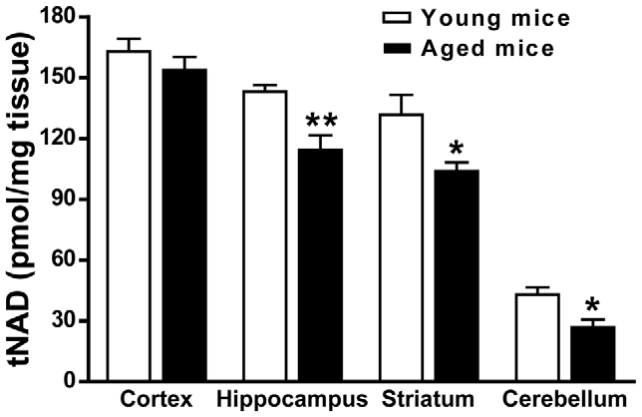
The intracellular level of tNAD in cortex, hippocampus, striatum and cerebellum. N = 6. Mean ± SEM. *P<0.05, compared with young mice, unpaired *t* test.

The tNAD level in hippocampus, striatum and cerebellum of aged mice were significantly lower than that of young mice, whereas there was no significant difference in cortex region (Fig. 2). In middle-aged mice brain, the tNAD level decreased only in cerebellum when compared with young mice brain (Figure S2A). The NADH level was about 1/6 of the NAD level in brain, and the NADH level remains largely the same in mice brain between young and middle age mice (Figure S2B).

### NAMPT distribution in brain

**Figure 3:**
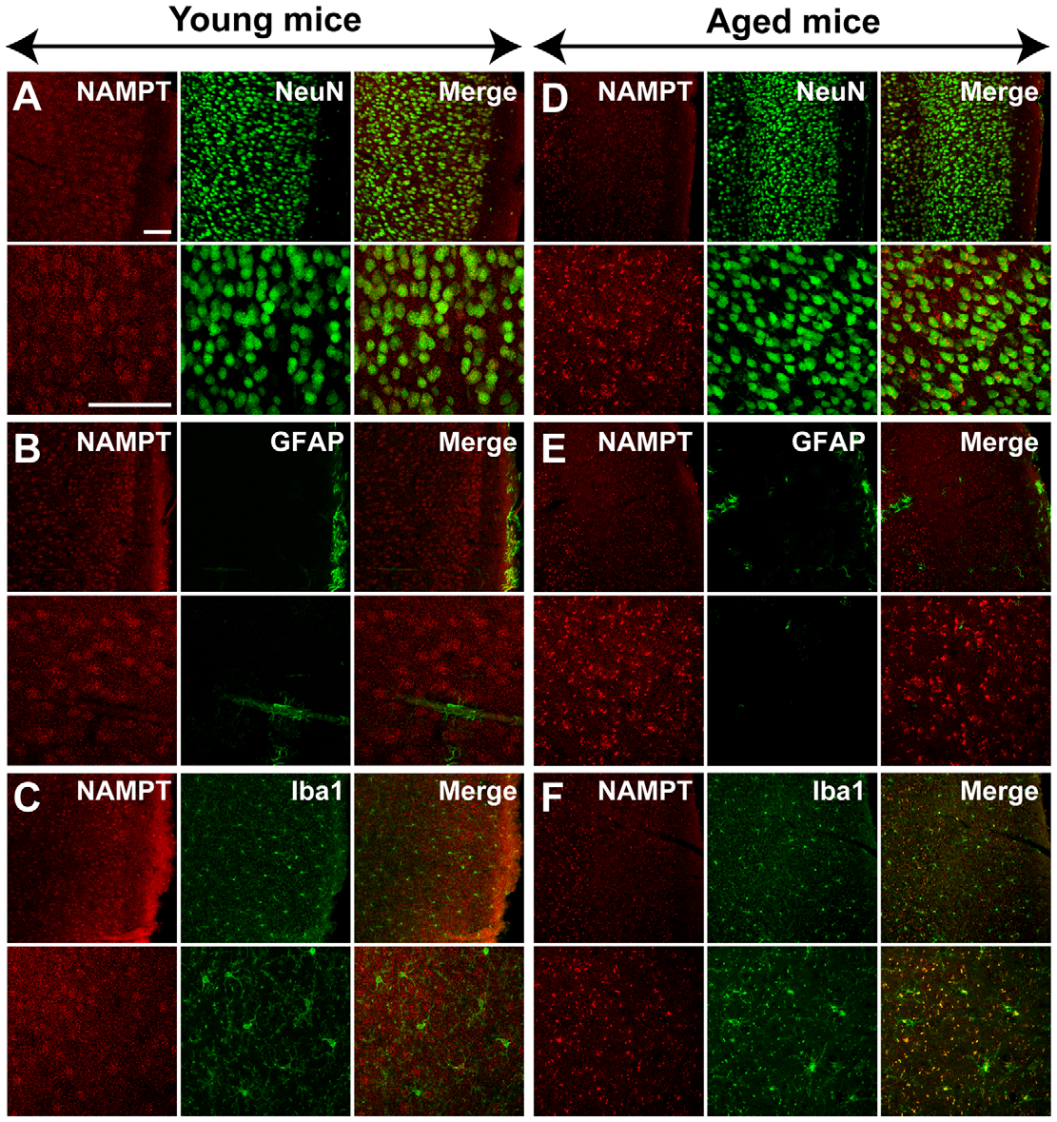
NAMPT distribution in cortex of young and aged mice. NAMPT is primarily distributed in NeuN-positive neurons (A and D), and is rare in GFAP-positive astrocytes (B and E). NAMPT is absent in Iba1-positive microglia of young mice but is expressed in this cell type of aged mice (C and F). Immunostaining was repeated on three mice. Scale bars = 100 µm.

**Figure 4:**
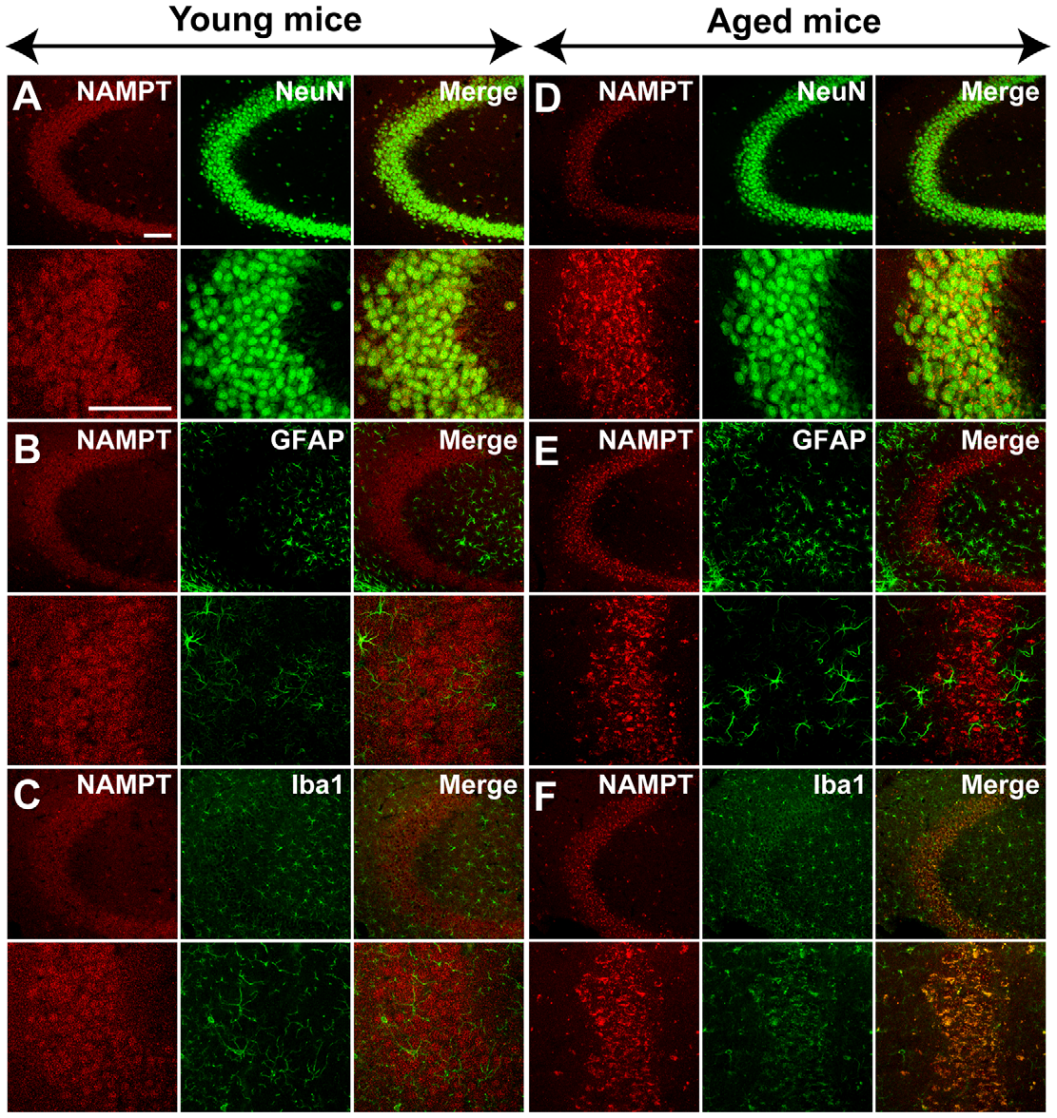
NAMPT distribution in hippocampus CA3 region of young and aged mice. NAMPT is primarily distributed in NeuN-positive neurons (A and D), and rare in GFAP-positive astrocytes (B and E). NAMPT is absent in Iba1-positive microglia of young mice but is present in the aged mice (C and F). Immunostaining was repeated on three mice. Scale bars = 100 µm.

**Figure 5:**
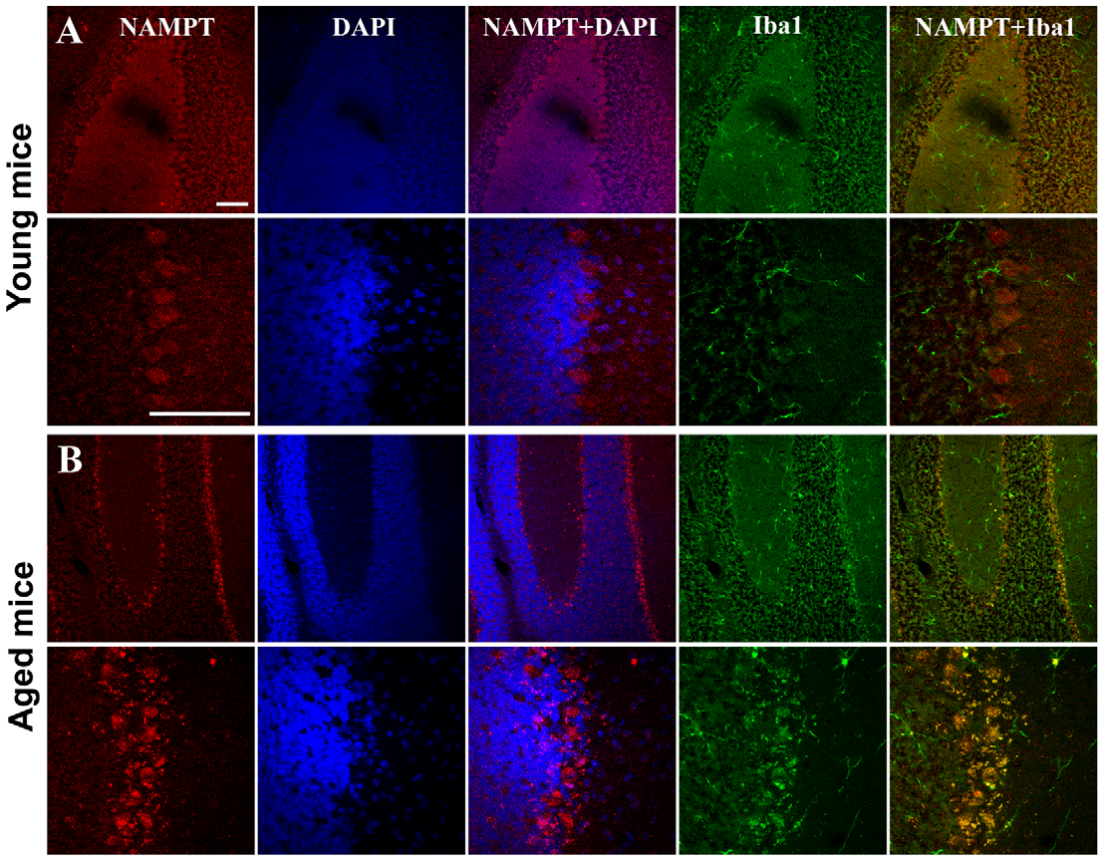
NAMPT distribution in the cerebellum of young (A) and aged mice (B). NAMPT is widely distributed in Pukinje cell, granular cell and the cells in the molecular layer. NAMPT is absent in Iba1-positive microglia of young mice but is present in of the aged mice. Immunostaining was repeated on three mice. Scale bars = 100 µm.

In young mice, immunoreactivity for NAMPT was primarily localized in NeuN-positive neurons in the cortex and hippocampus CA3 region (Fig. 3A and 4A), but rarely in GFAP-positive astrocytes (Fig. 3B and 4B) and Iba1-positive microglia cells (Fig. 3C and 4C). NAMPT was also found expressed in Purkinje cells, granule cells and cells in molecular layer of cerebellum (Fig. 5A).

In aged mice, NAMPT was also found expressed in neurons (Fig. 3D, 4D and 5B). In addition, it was found highly expressed in microglia cells (Fig. 3F, 4F and 5B), but not in astrocytes (Fig. 3E and 4E).

### FK866 injured primary cultured neurons

**Figure 6:**
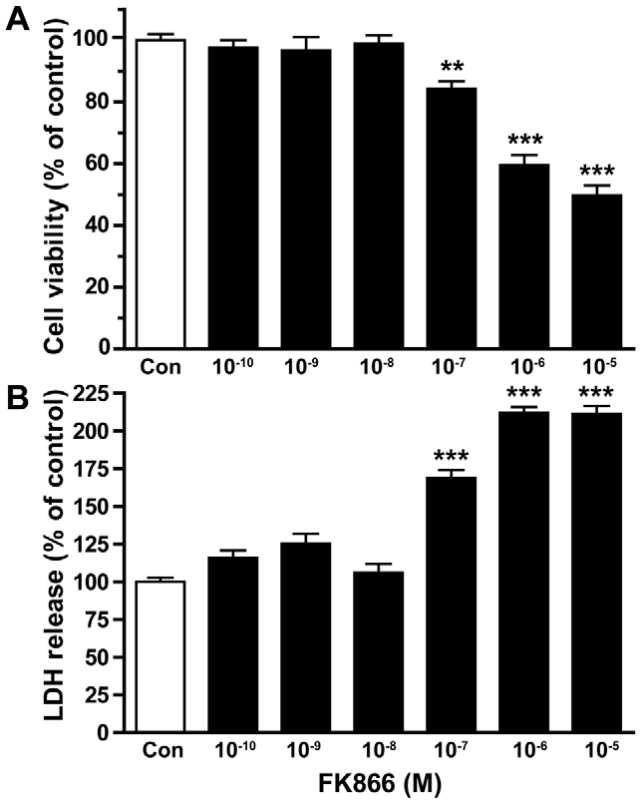
Effect of FK866 on cultured rat neuron viability. A) using MTT assay, cell viability was assed at 72 h after the treatment of FK866. B) LDH release was determined at 72 h after the treatment of FK866. N = 12. **P<0.01, ***P<0.001, compared with control, One-way ANOVA.

FK866, a potent inhibitor of NAMPT, was applied to the primary cultured rat cortex neurons, which would lower the intracellular NAD level. 72 h after FK866 application, the cell viability decreased, as determined by MTT assay (Fig. 6A), while release of LDH increased, proportional to the concentration of FK866 administered (Fig. 6B).

### NAMPT enhanced OGD-induced bEnd.3 cell injury

**Figure 7:**
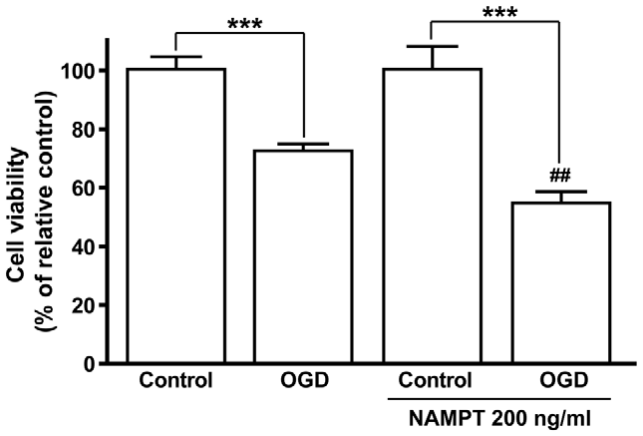
Effect of NAMPT treatment on OGD-induced injury in bEnd.3 cells. N = 6. ^##^ *P*<0.01, compared with OGD, One-way ANOVA.

Continuous application (5 generations and for a total of 15 days) of 200 ng/ml recombined NAMPT to vascular endothelia bEnd.3 cells aggravated OGD 1.5 h induced cell damages (Fig. 7).

## Discussion

In this study, we have found a region- and cell-specific change of NAMPT level upon aging — it decreased in brain while increases in serum; in brain, it increased in microglia but likely decreased in neuron. Accordingly, the level of tNAD, downstream product of NAMPT decreased in aged mouse brain. The declined in NAMPT protein level and possibly its enzymatic activity in aged brain may be responsible for gradual neuronal loss, whereas the increase of serum NAMPT may result in higher susceptibility of cerebral endothelial cell to ischemic-induced injury and inflammation. As such, we have shown for the first time, an age-dependent expression, distribution and activity pattern of NAMPT exist in the brain and serum, as well as the potential consequence of such alterations in aged brain.

The change of NAMPT level upon aging occurs in opposite direction in brain and serum – decreasing in brain while increasing in serum. The opposite trend of NAMPT level may be due to the fact that NAMPT in brain is mainly in the form of iNAMPT, while in serum it is eNAMPT. iNAMPT functions as an NAD biosynthetic enzyme and promotes cell survival [16], [29]. Decreasing NAMPT level in aged mice, likely iNAMPT, has also been reported in skeletal muscle [6], as well as in hippocampus and cerebellum [30]. The eNAMPT can both be an enzyme [31] and a pro-inflammatory cytokine [23], [32]. Elevated level of serum NAMPT, the major form of eNAMPT, has been reported in patients with age-related diseases such as diabetes, obesity, atherosclerosis, cancers and inflammation, implying an important role of NAMPT in these disease processes [20], [33]–[35]. Although aging *per se* is not a diseased state, elevated serum NAMPT may imply a less-than-healthy state.

The iNAMPT level in brain also changed in opposite directions for different types of cells during aging – increasing in microglia and likely decreasing in neurons. The expression of NAMPT in neuron but not in astrocyte and microglia in young mice brain is consistent with a previous report [3]. Here for the first time we have shown that NAMPT was also expressed in the neurons of cerebellum including Purkinje cells and granule cells. We found that NAMPT is absent in microglia of young mice brain but highly expressed in the microglia of aged mice brain, especially in hippocampus and cerebellum. Microglia is a type of inflammatory cell in central nervous system that possesses heightened reactivity in aged brain [26], while NAMPT has been shown to play a vital role in regulating peripheral inflammatory cells including macrophages [36], neutrophils [37] and lymphocyte [38]. Recently, it was reported that FK866, a NAMPT inhibitor, may inhibit the activation of microglia after spinal injury [39]. As such, the high level of NAMPT in microglia of aged mice indicates that NAMPT is involved in microglia activation in aged brain. Our results also suggested that NAMPT expression in neuron likely declined in aged mice brain, as the total NAMPT expression level decreased in aged brain, while the cellular distribution of NAMPT in aged brain became broadened. As NAMPT participates in the process of energy metabolism, the decreasing NAMPT in neuron and increasing NAMPT in microglia may be one of the causes for declining neuronal activity declined and elevated microglia activity in aged brain.

Consistent but not synchronized with the reduction of NAMPT in aged brain, tNAD level also decreased. The decrease in NAMPT and tNAD levels showed different brain-region specificity — upon aging NAMPT level significantly decreased in cortex and hippocampus but remained constant in striatum and cerebellum, while tNAD level significantly decreased in hippocampus, striatum and cerebellum but remained unchanged in cortex. The decrease in tNAD level in hippocampus and cerebellum but not in cortex upon aging is consistent with a previous report [30]. NAD reduction has also been reported in heart, lung, liver and kidney of aged rat [40]. The discrepancy between tNAD and NAMPT alteration upon aging may be due to the fact that NAD level was not only determined by the enzymatic activity and expression level of NAMPT [1], [41], but also by the consumption of NAD [42], [43] during energy metabolism [25] and by NAD-dependent enzyme, such as histone deacetylase sirtuins poly(ADP-ribose) polymerase 1 (PARP-1). Thus, age dependent increase in DNA damage and consequent over-activation of PARP-1 and situins would consume a large amount of NAD [40]. We hypothesize that the low activity of cortex neurons may result in low consumption of NAD, so that the NAD level remains constant even though NAMPT expression level decreases. On the other hand, the large amount of Iba-1 positive microglia in aged mice cerebellum may be responsible for the increased consumption of NAD and the decreased NAD level, while NAMPT level remains constant.

Finally, our study showed that both decrease of iNAMPT/NAD in brain and increase of eNAMPT in blood serum upon aging may cause age-dependent brain diseases and/or disorders. We found that the depletion of intracellular NAD by FK866 caused neuronal death, which imply that intracellular NAD is important for neuron survival. This is consistent with previous reports that iNAMPT and NAD is protective against neuron degeneration [30] and ischemic injury [2], [3]. The neuronal death induced by FK866 could be a result of the inhibition of mitochondrial function, because it was recently reported that inhibition of NAMPT decreased intracellular NAD and in turn inhibited mitochondrial function [44]. Importantly, we have found that the mouse cerebral vascular endothelial cells treated with recombinant NAMPT became more susceptible to ischemic injury. The injurious effect of eNAMPT on endothelial cell may be due to the fact that eNAMPT can generate inflammation and oxidative responses [20], [22], [23] and lipid raft redox [45]. As high serum NAMPT level was found in the patients with diabetes [33] and obesity [17], the enhancement of ischemic injury in endothelial cells by NAMPT may in part explain that patients with diabetes and obesity are more susceptible to stroke attack [46].

In summary, we have shown for the first time how the expression level and distribution of NAMPT in brain and serum change upon aging. These changes could be partly responsible for neuron loss and cerebral vascular endothelial dysfunctions, the hallmarks of aging. The changes may also be responsible for the onset of microglia-mediated neuro-inflammation upon aging. Hence, our findings suggest that NAMPT could be a regulatory factor in aging and age-related brain diseases.

## Materials and Methods

### Ethics Statement

All procedures were carried out in accordance with the recommendations in the Guide for the Care and Use of Laboratory Animals of the National Institutes of Health. The protocol was approved by Institutional Review Boards of Zhejiang University School of Medicine. All animal was deeply anesthetized under 10% chloral hydrate before sacrifice, and all efforts were made to minimize suffering.

### Animals

Young (2 months old, n = 31), middle-aged (10 months old, n = 12) and aged (16∼18 months old, n = 20) female ICR mice were purchased from Laboratory Animal Center of Zhejiang University (Zhejiang, PR China). Mice were housed in a temperature-conditioned room (22±1°C) under a 12-h light/dark cycle (light off from 18:00 to 6:00), and were given free access to food and water. Sprague-Dawley rats born within 12 h (n = 6) were purchased from Laboratory Animal Center of Zhejiang Academy of Medical Science, China.

### Cells and cell culture

Mouse-derived cerebral endothelial cells (bEnd.3) were purchased from the Institute of Cell Biology, Chinese Academy of Sciences (Shanghai, China). Cells were cultured in RPMI media 1640 medium (Gibco, Paisley, United Kingdom) supplemented with 10% heat inactivated fetal calf serum (FCS; Gibco) and 100 U/ml penicillin and streptomycin, at 37°C in a humidified atmosphere containing 95% air and 5% CO_2_.

Cortical neurons were prepared from the brains of Sprague-Dawley rats born within 12 h, and were plated at a density of 10^5^ cells per well in 96-well plates (Falcon, Franklin Lakes, NJ, USA) coated with 0.1 g/L poly-L-lysine (Adrich-Sigma, St Louis, MO, USA). Cells were grown in the plating medium containing 80% high-glucose DMEM, 10% fetal bovine serum, 10% horse serum, 2 mM glutamine and 100 U/mL penicillin/streptomycin (Gibco, Grand Island, NY, USA) for 24 h. The plating medium was changed to feeding medium containing 95% high-glucose DMEM, 5% horse serum, 2 mM glutamine, 100 U/mL penicillin/streptomycin, 0.01% N2 and 0.04% B27 (Gibco). The day of plating was identified as day-in-vitro 0 (DIV 0). On DIV 3, 10 µM cytosine arabinoside (Sigma-Aldrich, St. Louis, USA) was added to prevent the proliferation of non-neuronal cells. Half of the feeding medium was changed every 3 days, and cultures were maintained at 37°C until DIV 10 in a humidified atmosphere (5% CO_2_ and 95% air).

### Western blotting

100–200 µl blood was obtained and serum was collected, mice were then sacrificed and the cerebral cortex, hippocampus, striatum and cerebellum were quickly dissected on ice. Serum and brain samples were stored at −70°C until use. The brain samples were homogenized in Cell and Tissue Protein Extraction Solution (Kangchen Biotechnology, Shanghai, China). The homogenate was centrifuged at 14,000 g at 4°C for 30 min, and the supernatant was used. Protein concentrations were determined using Bio-Rad protein assay (Bio-Rad Lab, Hercules, CA, USA). The brain and serum samples were loaded for Western blotting and detected with respective antibodies. The primary antibodies included affinity-purified rabbit anti-NAMPT antibody (1∶1500; Bethyl, Japan) and mouse monoclonal antibody against glyceraldehyde-3-phosphate dehydrogenase (GAPDH, 1∶5000; Kangchen, China). The secondary antibody was IRDye 700-conjugated affinity-purified anti-rabbit IgG (1∶3000) and IRDye 800-conjugated affinity-purified anti-mouse IgG (1∶5000; Rockland Immunochemicals, Gilbertsville, PA, USA). The immunoblotting was analyzed by an Odyssey Fluorescent Scanner (LI-COR; Bioscience, Lincoln, NE, USA) and quantified using BIORAD Quantity One software (Bio-Rad, Hercules, CA, USA). The expression of NAMPT in brain are expressed as the ratio of NAMPT/GAPDH, while the expression of NAMPT in serum are expressed as the ratio of NAMPT/Ponceau staining according to a previous report [3].

### Immunohistochemistry

To visualize the cellular localization of NAMPT in brain, double immunofluorescence was applied onto floating 30 µm-thick brain sections. The sections were permeabilized in PBS with 0.3% Triton X-100 and 10% donkey serum for 30 min at room temperature. After wash, each section was incubated overnight at 4°C with the mixture of a rabbit antibody against NAMPT (1∶1000) together with a mouse monoclonal antibody against NeuN (1∶200), GFAP (1∶600), or Iba1 (1∶600). After wash, the sections were incubated with a mixture of Cy3-conjugated goat anti-rabbit IgG (1∶200) and FITC-conjugated goat anti-mouse IgG (1∶100), or a mixture of Cy3-conjugated donkey anti-rabbit IgG (1∶200) and FITC-conjugated donkey anti-goat IgG (1∶200). The sections were observed under a Fluoview FV1000 confocal microscope (Olympus) after sealed with anti-fading Permount medium (ProLong® Gold Antifade Reagent).

### tNAD and NADH quantification

The concentrations of tNAD and NADH in brain tissues were detected using a NAD^+^/NADH Quantification Kit (Bio Vision, K337-100) according to the manual. Briefly, fresh neocortex, striatum, hippocampus and cerebellum samples were homogenized in NADH/NAD extraction solution, and tNAD level was measured in the presence of NAD cycling mix and NADH developer. NADH was measured in the presence of NAD cycling mix but no NADH developer, and NAD was decomposed before the measurement by heating to 60°C for 30 min. The resulting optical density was read at 450 nm every 30 minutes for 4 hours. The tNAD and NADH levels were expressed as pmol/mg tissue.

### Oxygen-glucose deprivation (OGD)

The bEnd.3 cells were continuously applied with 200 ng/ml recombinant NAMPT (continuously applied for 5 generations, for total 15 days), then rinsed twice and incubated in Earle's solution without glucose and then the cells were introduced into an anaerobic chamber filled with 95% N_2_ and 5% CO_2_ at 37°C for 1.5 h. The control cells were incubated in Earle's solution without glucose and kept in a humidified atmosphere containing 5% CO_2_ and 95% air for 1.5 h.

### Cell viability and LDH release assay

MTT assay was used to assess cell viability. Cells cultured in 96-well plates were incubated with 100 µl MTT solution (0.5 mg/ml thiazolyl blue tetrazolium bromide in PBS) for 2 h at 37°C. Then, MTT solution was carefully removed, 100 µl DMSO were added to each well and incubated for 10 min. The absorbance was read at 490 nm in a plate reader (Elx800, Bio-TEK Instrument USA).

Using an LDH detection kit (Jiancheng Bioengineering Institute, Nanjing, China), the activity of LDH released into the medium was measured to determine cell death. After treatment, an aliquot of the medium was mixed with NAD and lactate solution, and the LDH product was measured at 450 nm (OD_450_) in a plate reader.

### Statistical analysis

Data were expressed as means ± S.E.M.. The differences between groups were evaluated with one-way ANOVA. GraphPad Prism version 4.0 (GraphPad Software, San Diego, CA) was used for statistical analysis. *P*<0.05 was considered statistically significant.

## Supporting Information

Figure S1 **Immunoblotting analysis for NAMPT expression in blood serum and brain regions for young and middle-aged mice.** Upper panel, representative immunoblots of NAMPT and GAPDH expression in cortex, hippocampus, striatum and cerebellum, or immunoblots of NAMPT expression and ponceau staining in blood serum. Lower panel, statistical analyses of NAMPT expression. N = 8. Mean ± SEM. **P<0.01, compared with young mice, unpaired *t* test.
(DOCX)

Figure S2 **The intracellular level of tNAD (A) and NADH (B) in brain regions for young and middle-aged mice.** N = 4. Mean ± SEM. *P<0.05, compared with young mice, unpaired *t* test.
(DOCX)
