# Supplementary material for: Nicotinamide Phosphoribosyltransferase May Be Involved in Age-Related Brain Diseases
Source: PLoS One. 2012 Oct 11;7(10):e44933. doi: 10.1371/journal.pone.0044933 (PMC3469563; doi:10.1371/journal.pone.0044933)
Supplement: Figure S1 — Immunoblotting analysis for NAMPT expression in blood serum and brain regions for young and middle-aged mice. Upper panel, representative immunoblots of NAMPT and GAPDH expression in cortex, hippocampus, striatum and cerebellum, or immunoblots of NAMPT expression and ponceau staining in blood serum. Lower panel, statistical analyses of NAMPT expression. N = 8. Mean ± SEM. **P<0.01, compared with young mice, unpaired t test. (DOCX) [file pone.0044933.s001.docx]

**
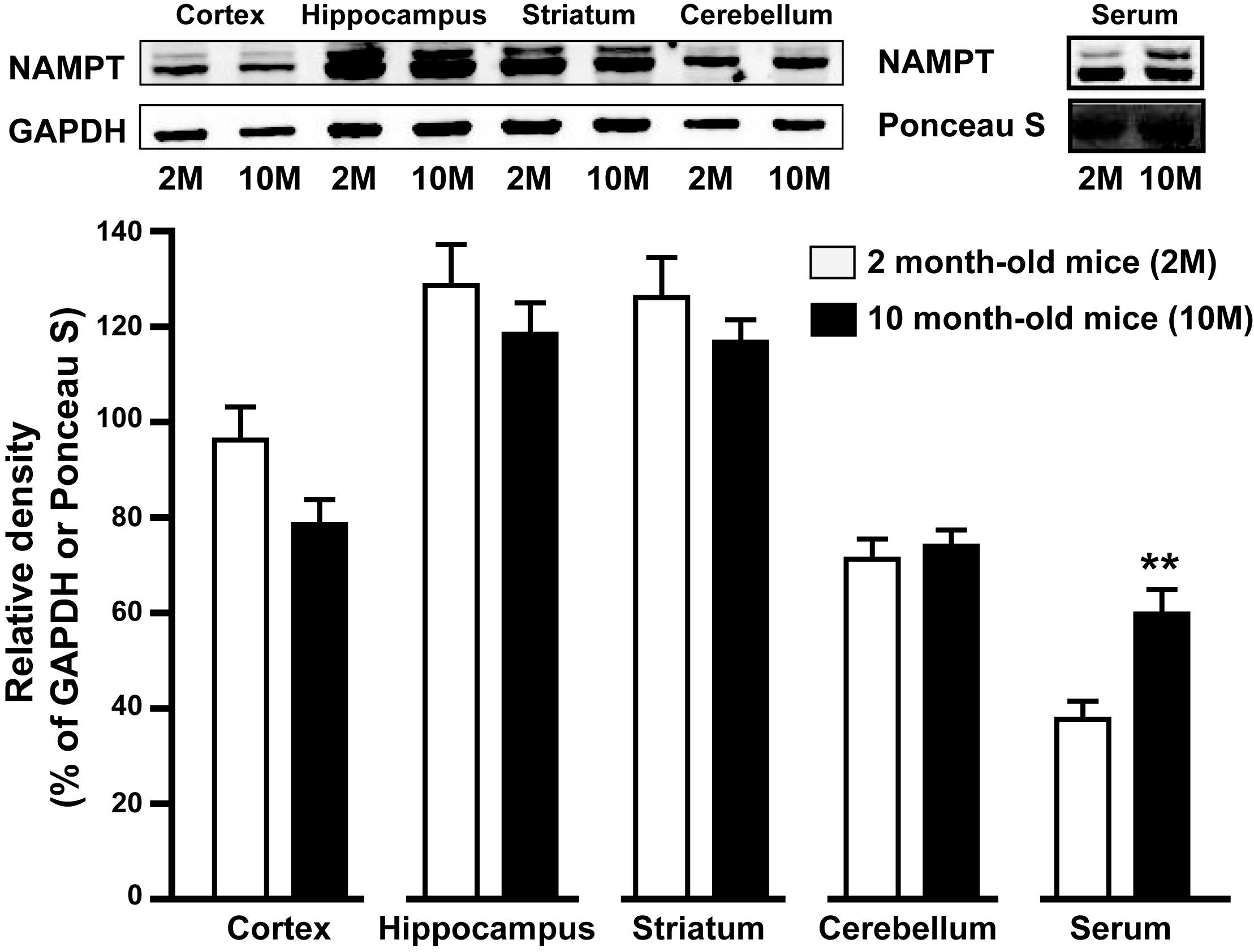
**

**Figure S1.**  **Immunoblotting analysis for NAMPT expression in blood serum and brain regions for young and middle-aged mice.** Upper panel, representative immunoblots of NAMPT and GAPDH expression in cortex, hippocampus, striatum and cerebellum, or immunoblots of NAMPT expression and ponceau staining in blood serum. Lower panel, statistical analyses of NAMPT expression. N=8. Mean ± SEM. ***P*<0.01, compared with young mice, unpaired *t* test.
