# Supplementary material for: Nicotinamide Phosphoribosyltransferase May Be Involved in Age-Related Brain Diseases
Source: PLoS One. 2012 Oct 11;7(10):e44933. doi: 10.1371/journal.pone.0044933 (PMC3469563; doi:10.1371/journal.pone.0044933)
Supplement: Figure S2 — The intracellular level of tNAD (A) and NADH (B) in brain regions for young and middle-aged mice. N = 4. Mean ± SEM. *P<0.05, compared with young mice, unpaired t test. (DOCX) [file pone.0044933.s002.docx]

**
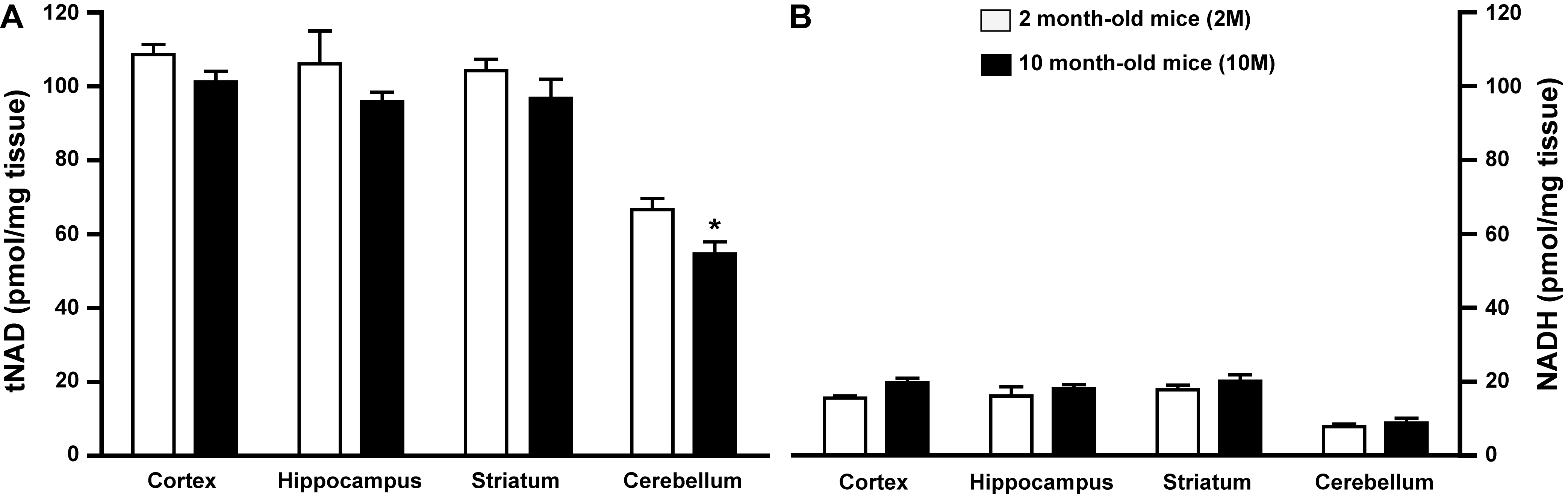
**

**Figure S2.** **The intracellular level of tNAD (A) and NADH (B) in brain regions for young and middle-aged mice.** N=4. Mean ± SEM. **P*<0.05, compared with young mice, unpaired *t* test.
